# Anticancer Effects of* Salvia miltiorrhiza* Alcohol Extract on Oral Squamous Carcinoma Cells

**DOI:** 10.1155/2017/5364010

**Published:** 2017-01-29

**Authors:** Wen-Hung Wang, Kuo-Yu Hsuan, Ling-Ya Chu, Chia-Ying Lee, Yu-Chang Tyan, Zong-Shiow Chen, Wan-Chi Tsai

**Affiliations:** ^1^Department of Otolaryngology, Cathay General Hospital, Taipei City 106, Taiwan; ^2^Department of Otolaryngology, Sijhih Cathay General Hospital, New Taipei City 221, Taiwan; ^3^School of Medicine, Fu-Jen Catholic University, New Taipei City 242, Taiwan; ^4^Department of Hemato-Oncology, Chi-Mei Medical Center, Tainan City 710, Taiwan; ^5^Department of Medical Laboratory Science and Biotechnology, Kaohsiung Medical University, Kaohsiung 807, Taiwan; ^6^Department of Medical Imaging and Radiological Sciences, Kaohsiung Medical University, Kaohsiung 807, Taiwan; ^7^Center for Infectious Disease and Cancer Research, Kaohsiung Medical University, Kaohsiung 807, Taiwan; ^8^Graduate Institute of Medicine, College of Medicine, Kaohsiung Medical University, Kaohsiung 807, Taiwan; ^9^Institute of Medical Science and Technology, National Sun Yat-Sen University, Kaohsiung 804, Taiwan; ^10^Department of Medical Research, Kaohsiung Medical University Hospital, Kaohsiung 807, Taiwan; ^11^Institute of Cosmetic Science, Chia-Nan University of Pharmacy and Science, Tainan City 717, Taiwan

## Abstract

Researchers have reported significant effects from Danshen (*Salvia miltiorrhiza*) in terms of inhibiting tumor cell proliferation and promoting apoptosis in breast cancer, hepatocellular carcinomas, promyelocytic leukemia, and clear cell ovary carcinomas. Here we report our data indicating that Danshen extracts, especially alcohol extract, significantly inhibited the proliferation of the human oral squamous carcinoma (OSCC) cell lines HSC-3 and OC-2. We also observed that Danshen alcohol extract activated the caspase-3 apoptosis executor by impeding members of the inhibitor of apoptosis (IAP) family, but not by regulating the Bcl-2-triggered mitochondrial pathway in OSCC cells. Our data also indicate that the extract exerted promising effects in vivo, with HSC-3 tumor xenograft growth being suppressed by 40% and 69% following treatment with Danshen alcohol extract at 50 and 100 mg/kg, respectively, for 34 days. Combined, our results indicate appreciable anticancer activity and significant potential for Danshen alcohol extract as a natural antioxidant and herbal human oral cancer chemopreventive drug.

## 1. Introduction

Oral cancer, the sixth most common cancer worldwide [1], is ranked third in South Central Asia [2]. Over 90% of all identified oral cavity cancers are invasive oral squamous cell carcinomas (OSCCs) [3–5] whose relative five-year survival rates range from 50% to 60% [3, 6]. Primary treatments are surgery, radiation therapy, and chemotherapy, all of which have severe side effects and poor response rates [7]. Herbal medicine is one of the most frequently used alternative therapies, several of which have been used alongside conventional treatment regimens [8, 9]. The use of medicinal herbs or their extracts is currently attracting attention as a promising chemopreventive strategy. One agent receiving particularly strong interest is Danshen (*Salvia miltiorrhiza*), one of the most widely and longest-used herbal medicines for numerous maladies throughout Asia. Previous studies indicate that Danshen has anti-inflammatory [10–12], antioxidant [12, 13], and blood circulation-improvement characteristics [10, 14] and some researchers have suggested that it has therapeutic advantages for several cancer types, including breast, prostate, and lung cancer [15–18]. However, few efforts have been made to test the effectiveness of Danshen extracts to treat oral cancer.

For the present study, we looked at the antioxidant activity of different Danshen extracts and their in vivo effects on cell proliferation and tumorigenesis in two human OSCC cell lines, HSC-3 and OC-2. Our results indicate that 95% crude Danshen alcohol extract exhibited the highest level of free radical scavenging and antitumor activity of all the extracts we examined. Further, we observed that Danshen alcohol extract induced apoptosis by regulating apoptosis protein family inhibition, with the exception of the Bcl-2-driven apoptosis pathway in OSCC cells. We therefore suggest that Danshen alcohol extract has significant potential for the treatment of oral squamous cell carcinomas.

## 2. Materials and Methods

### 2.1. Plant Extract Preparation

*Salvia miltiorrhiza* were purchased from Chuang Song-Zong Pharmaceutical Co., Ltd., in Kaohsiung City, Taiwan. Pieces of* Salvia miltiorrhiza* were isolated, washed, cut into small sections, soaked in solutions consisting of either double-distilled water (ddH_2_O), 95% ethanol, or 1 : 1 water/ethanol, and extracted by heating under reflux. Extracts were concentrated and strained through 0.45 *μ*m filters. All extraction processes were repeated twice. Extracts were powdered by freeze-drying and stored at −20°C.

### 2.2. 2,2-Diphenyl-1-picrylhydrazyl (DPPH) Assay

The antioxidant capacities of various Danshen extracts were determined using the DPPH radical scavenging method described in [19]. Briefly, 100 *μ*L concentrations of the various extracts in ethanol were added to 750 *μ*L of a 0.0025% ethanol DPPH solution. After 30 min of incubation at room temperature, absorbance was read against a blank at 517 nm. DPPH is a purple-colored stable free radical that changes to a yellowish diphenylpicrylhydrazine when reduced. The water-soluble vitamin E analogue 6-hydroxy-2,5,7,8-tetramethylchroman-2-carboxylic acid (Trolox) was used as a positive control. Inhibition ratios were calculated using the formula [(Ac − As)/Ac] × 100%, with Ac denoting control absorbance and As test sample absorbance. Extract concentrations providing 50% inhibition (IC_50_) were calculated using graphs plotting inhibition percentage against extract concentration [20].

### 2.3. 2,2′-Azinobis(3-ethylbenzothiazoline-6-sulphonic Acid) (ABTS) Assays

The free radical scavenging capabilities of the essential oils were determined using ABTS radical cation decolorization assays as described in [20]. ABTS radical cations were produced by reacting ABTS solution with 2.45 mM potassium persulfate, and allowing the mixture to stand in darkness at room temperature for 12–16 h. Incubation mixtures (5 mL total volumes) contained 0.54 mL of ABTS radical cations, 0.5 mL of phosphate buffer, and varying concentrations of individual extract. Appropriate solvent blanks were run with each assay. Absorbance was read by a spectrophotometer at 734 nm and compared with the Trolox control [20].

### 2.4. Cell Viability Analyses

The effects of Danshen extracts on cell viability were assessed using 3-(4,5-dimethylthiazol-2-yl)-2,5-diphenyl-2H-tetrazolium bromide (MTT) assays (six replications). Cells (3 × 10^3^) were seeded in 10% FBS-supplemented growth medium in 96-well plates for 24 h and exposed to indicated concentrations (2, 5, 10, 25, or 50 *μ*g/mL) of Danshen extract in the same medium for 24, 48, or 72 h. Control cells were treated with DMSO or a ddH_2_O vehicle at the same concentrations. Following treatment, medium was removed and replaced with 200 *μ*L of 0.5 mg/mL MTT, after which cells were incubated in a CO_2_ incubator at 37°C for 2 h. After removing supernatant from each well, reduced MTT dye was solubilized in DMSO (200 *μ*L/well). Absorbance was determined at 595 nm using a plate reader.

### 2.5. Caspase-3 Activity Assays

Caspase-3 activity was determined using an FITC Active Caspase-3 Apoptosis Kit (BD Biosciences) according to manufacturer instructions. Cells were treated with DMSO or indicated concentrations of Danshen extract (10, 25, or 50 *μ*g/mL). After 48 h, cells were collected and incubated with Cytofix/Cytoperm solution at 4°C for 20 minutes; solution was removed by centrifugation at 3000 rpm for 5 min. Cells were then incubated with FITC-conjugated monoclonal rabbit anti-active human-caspase-3 antibody for 30 minutes at room temperature. Cells were washed twice with PBS, and 500 *μ*L of Perm/Wash buffer was added prior to flow cytometry.

### 2.6. Mitochondrial Membrane Potential

Mitochondrial membrane potential was quantified by flow cytometry using a MitoProbe JC-1 Assay Kit (Life Technologies). Cells were treated with Danshen extract (10, 25, or 50 *μ*g/mL), DMSO (control), or 50 *μ*M carbonyl cyanide 3-chlorophenylhydrazone (CCCP) as a positive control. After 48 h, cells were collected and incubated with 200 *μ*M JC-1 dye for 30 minutes at 37°C. Cells were centrifuged at 1300 rpm for 5 minutes to remove supernatant and resuspended in 1 mL PBS prior to flow cytometry.

### 2.7. Protein Extraction and Western Blot Analyses

Apoptosis biomarkers were assessed by Western blotting. Treated cells were washed in PBS, resuspended in sodium dodecyl sulfate (SDS) sample buffer, sonicated for 5 sec, and boiled for 5 min. After brief centrifugation, equal amounts of total protein from each sample were fractionated by sodium dodecyl sulfate polyacrylamide gel electrophoresis (SDS-PAGE) and transferred to a polyvinylidene difluoride membrane that was washed three times with Tris-buffered saline (TBS) containing 0.05% Tween 20 (TBST). After blocking with TBST containing 5% nonfat milk for 60 min, the membrane was incubated overnight with an appropriate primary antibody at 1 : 1000 dilution in TBST-5% low-fat milk at 4°C and then washed three more times with TBST. The membrane was probed with goat anti-rabbit or anti-mouse IgG-horseradish peroxidase conjugate (1 : 10000) for 1 h at room temperature and washed three more times with TBST. Hybridized immunocomplexes were detected with Renaissance Chemiluminescence Reagent Plus (NEN Life Science Products, Boston).

### 2.8. Animal Experiments

Male BALB/c NU mice (6–8 weeks old) (purchased from BioLASCO Co., Ltd., Taiwan) were subcutaneously injected with HSC-3 cells (2 × 10^6^). As tumors became established, mice were randomly assigned to one of three groups and treated daily with either Danshen extract or DMSO (50 or 100 mg/kg) by intraperitoneal injection. Mouse weights and tumor volumes (length × width^2^ × 0.5) were measured daily. Mice were sacrificed when tumor volumes reached 2,000 mm^3^. Portions of each tumor were snap-frozen in liquid nitrogen and stored at −80°C until needed for Western blot analysis of relevant biomarkers. All experimental procedures were performed in accordance with protocols approved by the Institutional Laboratory Animal Care and Use Committee of Kaohsiung Medical University.

### 2.9. Statistical Analyses

All results are presented as mean ± SEM. Statistical analyses of control and treatment data were executed in the form of Student's *t*-tests, with significance defined as *p* < 0.05 in all cases.

## 3. Results and Discussion

### 3.1. Determination of Antioxidant Activity of Danshen Extracts

**Figure 1:**
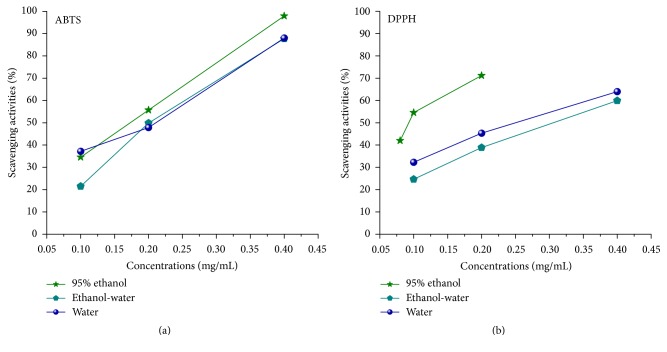
Radical scavenging activity for three Danshen extract types. Effect data for different concentrations of three Danshen extracts from free radical scavenging tests. (a) Assay data for scavenging of free radical ABTS with SC_50_ values of 0.197, 0.232, and 0.223 *μ*g/mL. (b) Data for DPPH with SC_50_ values of 0.094, 0.311, and 0.26 *μ*g/mL.

In mammalian systems, reactive oxygen species are produced naturally due to oxidative metabolism. In addition to contributing to a variety of physiological and biochemical lesions, free radicals can induce degenerative illnesses such as coronary artery disease and cancer [21, 22]. For this study we analyzed three types of Danshen extracts in terms of their antioxidant and radical scavenging capabilities. ABTS and DPPH assay data are presented in Figure 1. As shown, the scavenging ability of Danshen alcohol extract had significantly higher values (0.197 for ABTS and 0.094 for DPPH) compared to water/alcohol (0.232 for ABTS and 0.311 for DPPH) and water-only extracts (0.223 for ABTS and 0.26 for DPPH). In comparison, the SC50 values of Trolox (a positive control) were only 0.048 and 0.022 for ABTS and DPPH scavenging, respectively. The data clearly indicate greater antioxidant potency for Danshen alcohol extract.

### 3.2. Cytotoxicity of Danshen Alcohol Extract in OSCC Cells

**Figure 2:**
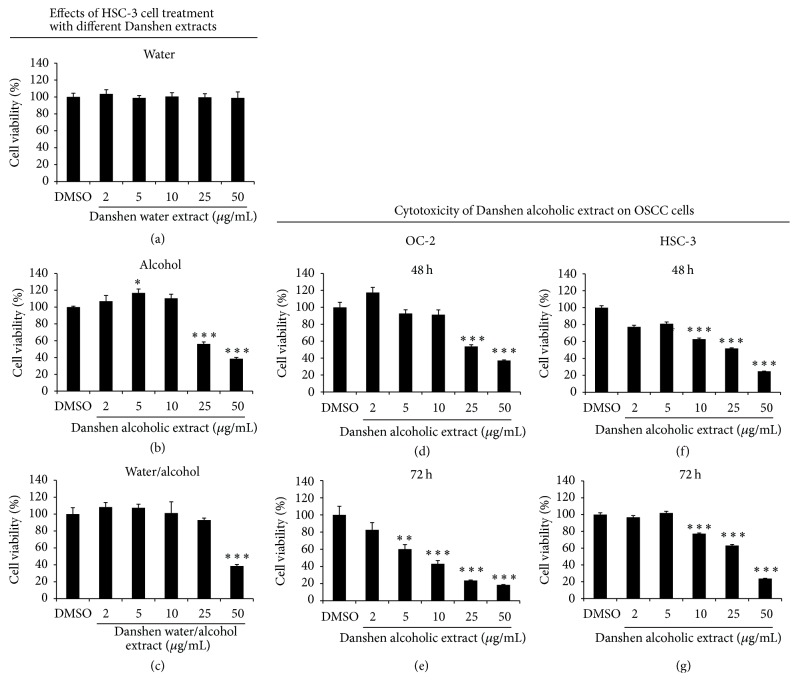
Cytotoxicity of different Danshen extracts on OSCC cells. (a–c) Cytotoxic effects of different Danshen extracts on HSC-3 cells at 24 h after treatment. (d, e) OC-2 and (f, g) HSC-3 cells were treated with 0, 2, 5, 10, 25, or 50 *μ*g/mL of Danshen alcohol extract for 48 or 72 h. MTT assay data are shown as mean ± SEM (*n* = 6). ^*∗*^*p* < 0.05; ^*∗∗*^*p* < 0.01; ^*∗∗∗*^*p* < 0.001.

Three different Danshen extraction methods were assessed in vitro using MTT assays to determine their antiproliferative capabilities against HSC-3 cells (Figures 2(a)–2(c)). Cells were treated with a Danshen extract (water, 95% alcohol, or a 1 : 1 mixture) at various concentrations. At 24 h after treatment, alcohol and alcohol/water extracts exhibited significantly stronger antiproliferative effects among the three types (IC_50_ values of 39.8 and 47.1 *μ*g/mL, resp.). Next, we attempted to verify Danshen alcohol extract cytotoxicity against HSC-3 and OC-2 cells. Our results indicate appreciable dose- and time-dependent inhibitory effects in both cell lines (Figures 2(d)–2(g)). IC_50_ values of 26.67 and 30.68 *μ*g/mL were observed 48 h after treatment for HSC-3 and OC-2 cells, respectively. We noted that these IC_50_ values exceeded that for 50 *μ*g/mL of normal oral keratinocytes (Figure S1; see Supplementary Material available online at https://doi.org/10.1155/2017/5364010).

### 3.3. Apoptosis Induction in Danshen Alcohol Extract-Treated HSC-3 Cells

**Figure 3:**
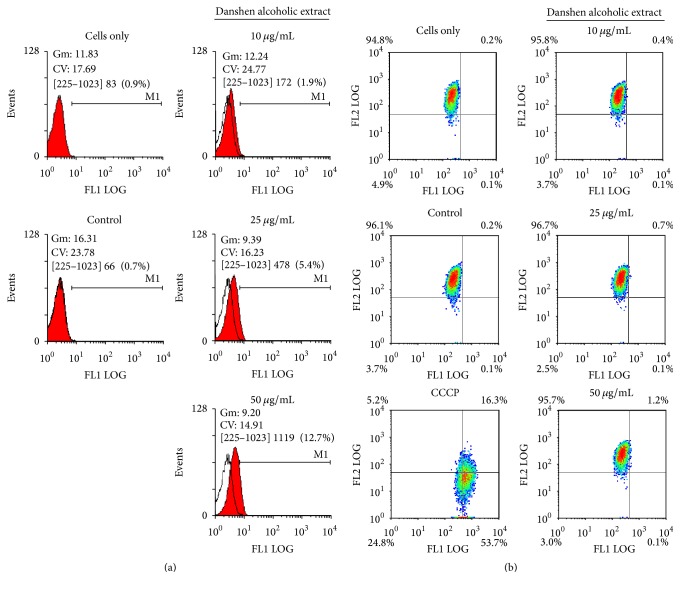
Danshen alcohol extract induces caspase-3 apoptotic pathway in HSC-3 cells. (a) Cells were treated with indicated concentrations of Danshen alcohol extract. Apoptosis was estimated using ITC Active Caspase-3 Apoptosis Assays. DMSO and camptothecin (Camp) served as a control and positive control, respectively, for caspase-3 activity. (b) Cells were incubated with different concentrations of Danshen alcohol extract. Mitochondrial membrane potential (ΔΨm) was measured by flow cytometry with JC-1 staining. DMSO and carbonyl cyanide 3-chlorophenylhydrazone (CCCP) served as a control and positive control, respectively.

**Figure 4:**
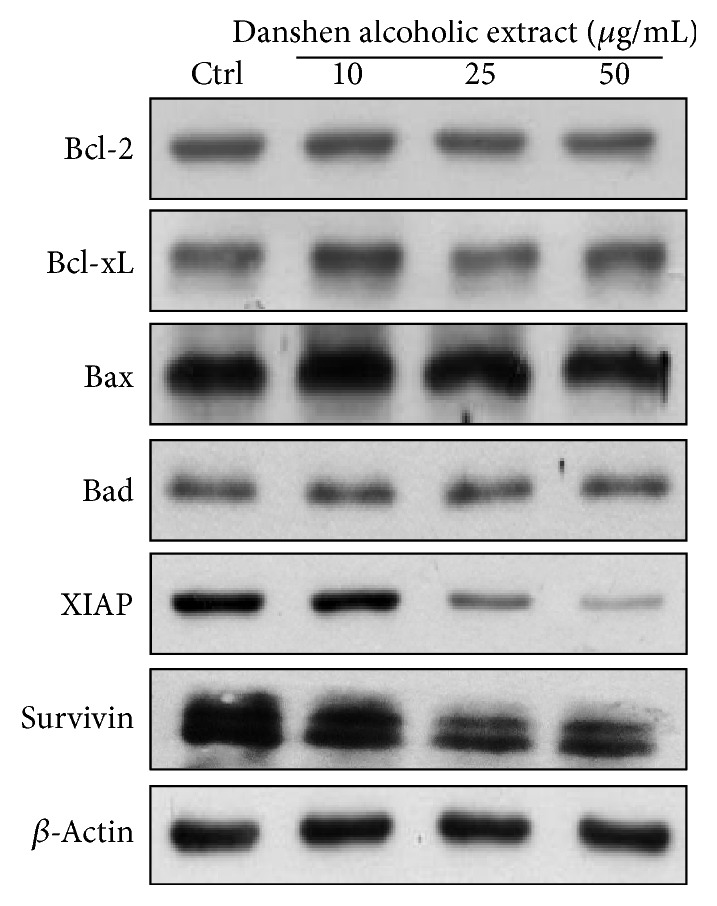
HSC-3 cells were analyzed by Western blotting 48 h following treatment with Danshen alcohol extract at concentrations of 10, 25, or 50 *μ*g/mL.

Cell apoptosis is triggered via intrinsic and extrinsic pathways. Intrinsic pathways are initiated by the loss of mitochondrial membrane potential (Δ*ψ*), leading to the release of cytochrome c from mitochondrial intermembranes and resulting in the formation of caspase activation platforms (apoptosomes) that trigger apoptotic protease cascades [23, 24]. For further characterization, we measured active caspase-3 (a marker for cells undergoing apoptosis) and found that it gradually increased 48 h following treatment with Danshen alcohol extract at 10, 25, or 50 *μ*g/mL (Figure 3(a)). To identify the upstream trigger of caspase-3 activation, we used cytometric analysis with JC-1 staining to evaluate apoptosis-related mitochondrial changes associated with Danshen alcohol extract and surprisingly found no change in detected Δ*ψ* (Figure 3(b)). This finding is consistent with our data for other apoptotic markers; levels of antiapoptotic proteins Bcl-2 and Bcl-xL and the proapoptotic proteins Bax and Bad [25, 26] remained relatively unchanged following HSC-3 cell treatment with Danshen alcohol extract (Figure 4). At the same time, we observed dramatic decreases in the expression of both XIAP and survivin, two members of the inhibitor of apoptosis protein (IAP) family. Combined, the data suggest that IAP family members, but not intrinsic apoptosis regulators, triggered the Danshen alcohol extract-induced apoptosis that we observed.

### 3.4. In Vivo Antitumor Growth Efficacy of Danshen Alcohol Extract

**Figure 5:**
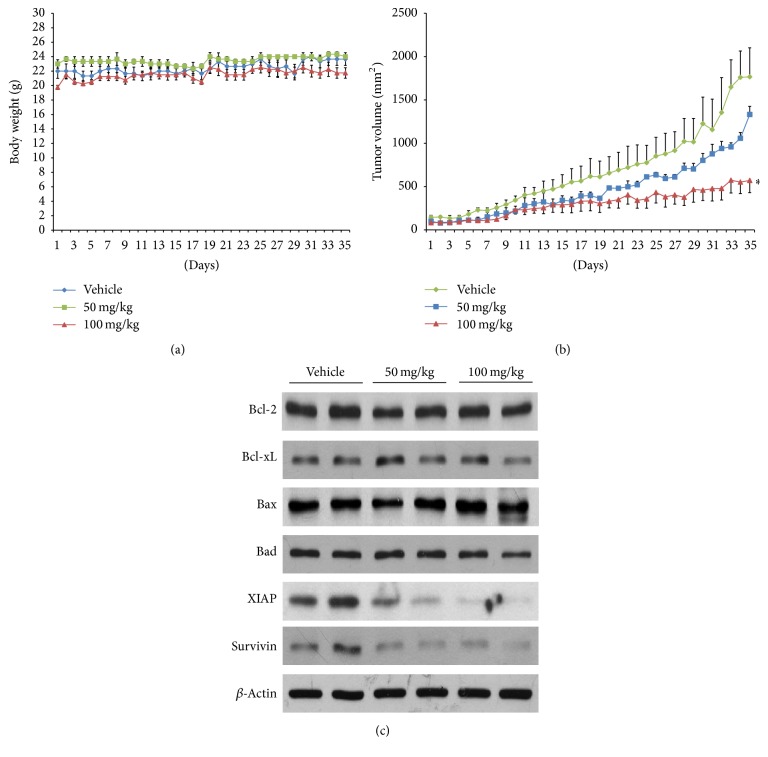
Effect of treatment with Danshen alcohol extract on tumor growth in BALB/c NU mice. (a) Body weights of mice from control, 50 mg/kg, and 100 mg/kg treatment groups. (b) Tumor volume data. Results are presented as mean ± SEM (*n* = 3). ^*∗*^*p* < 0.05 compared to vehicle control at the endpoint of the experiment. (c) Western blot analysis data for protein expression of Bcl-2, Bcl-xL, Bax, Bad, XIAP, and survivin, using tumor protein extracts from 2 mice in each group. *β*-actin was served as loading control.

To further clarify the clinical implications of Danshen alcohol extract, we examined its antitumor effects in vivo. Male BALB/c NU mice (6–8 weeks old) were given subcutaneous injections of oral squamous carcinoma HSC-3 cells, followed by daily intraperitoneal injections of Danshen alcohol extract at 50 and 100 mg/kg body weight for 34 days; control mice were treated with DMSO. Mouse body weights were recorded and tumor volumes monitored daily. As shown in Figure 5(a), no significant impacts on mouse body weights were observed over the 34-day injection period, which indicated that no overt signs of toxicity were noted in any of the treated mice. However, the mice treated with the Danshen alcohol extract had significantly smaller tumor volumes (1056.06 ± 66.64 mm^2^ and 552.02 ± 133.40 mm^2^ for mice treated with 50 mg/kg and 100 mg/kg, resp.; *p* < 0.01) (Figure 5(b)). Compared to control group mice (1761.11 ± 302.86 mm^2^), average tumor growth in mice treated with Danshen alcohol extract was reduced by 39.9% for the 50 mg/kg dosage group and 68.7% for the 100 mg/kg dosage group. To examine biological markers in vivo, we randomly selected tumor tissue taken from 2 mice in each group to examine protein expression and found that, similar to the in vitro data, treatment with Danshen alcohol extract resulted in the downregulation of XIAP and survivin but not Bcl-2 family members (Figure 5(c)).

### 3.5. Potential Mechanisms and Active Ingredients

Many studies of Danshen's antitumor potential have produced significant findings. Active components of Danshen include danshensu, tanshinones, and salvianolic acids, all of which have been shown to exert antioxidant, antimicrobial, anti-inflammatory, anticancer, and cardiovascular-protective effects [27–29]. These clinical effects are generally attributed to two major Danshen components: tanshinone IIA (Tan-IIA) and salvianolic acid B (Sal-B) [29–31]. According to one report, Tan-IIA is capable of inducing cell apoptosis and inhibiting cell proliferation in hepatocellular carcinomas [32], promyelocytic leukemia [33–35], erythroleukemia [34, 35], and ER-positive breast cancer cells [36]. Tan-IIA has also been shown to prevent cells from oxidant damage [37–39] and lipid peroxidation [40]. In oral cavities, the hyperexpression of cycloxygenase-2 (COX-2) increases the risk of developing head and neck cancers, but these risks are reduced by Sal-B [41]. Sal-B anticancer mechanisms involve the attenuation of OSCC cell growth by blocking COX-2 pathways, inhibiting angiogenesis, and inducing apoptosis [28]. Results from previous studies suggest that the anticancer effects of Danshen may be due to apoptosis modulation and angiogenesis regulation, in addition to its anti-inflammatory and antioxidative properties [28]. However, the mechanisms underlying its therapeutic potential remain unclear. This served as our motivation to analyze various Danshen extracts in terms of their antiproliferative activity and antioxidant effects and to specifically analyze Danshen alcohol extract in terms of its anticancer potential against human OSCC cells. Our data reveal high levels of radical scavenging and chemopreventive activity in vitro and in vivo.

Acknowledging that different types of Danshen might contain different component ratios that affect antiproliferative and antioxidant efficacy and due to the fact that weather, soil conditions, and other factors can affect the herb's therapeutic value, we also investigated the active ingredients and antioxidant effects of Danshen extracts from different sources and extraction methods. Specifically, we examined Taiwan- and China-grown Danshen samples extracted by water, 1 : 1 water/ethanol, and 95% ethanol. All extracts were found to be rich in Tan-IIA and Sal-B and to exhibit linear correlations between the two components and antioxidant capacity, especially Taiwan-grown samples extracted with alcohol (Tables S1–S3). The higher Tan-IIA and Sal-B content was the primary reason for using Taiwan-grown Danshen in this study. It is plausible to suggest that those major components significantly influenced the antioxidant activity of the Danshen extracts used in this research, thereby directly or indirectly affecting their antitumor capacities. Our findings are consistent with those from previous studies demonstrating the anticarcinogenic capacities of Tan-IIA and Sal-B via the prevention of oxidative damage [37].

Multiple studies have shown that caspase activation and the loss of mitochondrial membrane potential initiate apoptosis. However, some researchers have reported that mitochondria are not always involved as apoptotic stimuli [42, 43]. In the present study we also observed an apoptotic effect with no change in mitochondrial membrane potential. Caspase-3 activity is known to be inhibited by a group of proteins collectively labeled “inhibitors of apoptosis proteins.” We also looked for XIAP and survivin expression due to previous reports showing that they directly bind and inhibit caspase-3 [44, 45]. Survivin, a bifunctional protein that regulates cell division and suppresses apoptosis, is commonly expressed in normal tissue and overexpressed in several types of human cancers [20]. Survivin prevents cell apoptosis via the overexpression of procaspase-3 and the suppression of active caspase-3, thereby increasing tumor cell proliferation and decreasing chemotherapy response [44]. The complete removal of XIAP and survivin that we observed serves as evidence that Danshen alcohol extract induced cell death by mitigating apoptosis inhibition.

## 4. Conclusion

Danshen extracts, especially alcohol extract, exhibit appreciable antioxidant and antitumor activity. Our data indicate that Danshen alcohol extract induced OSCC cell apoptosis, inhibited XIAP, and activated caspase-3. We also found evidence that Danshen alcohol extract may suppress HSC-3 tumor growth in vivo without notable side effects such as body weight loss. Combined, our results indicate appreciable anticancer activity from Danshen alcohol extract and potential for it to be applied as a natural antioxidant and herbal human oral cancer chemopreventive drug.

## Supplementary Material

Gingival tissue from healthy outpatients with the patients' consent was used as a source of NOK, which were maintained in Keratinocyte serum-free medium (Invitrogen, Carlsbad, CA) and used within ﬁve passages.
