# Corrigendum to “Anticancer Effects of* Salvia miltiorrhiza* Alcohol Extract on Oral Squamous Carcinoma Cells”

**DOI:** 10.1155/2017/4695951

**Published:** 2017-05-11

**Authors:** Wen-Hung Wang, Yu-Hsuan Kuo, Ling-Ya Chu, Chia-Ying Lee, Yu-Chang Tyan, Zong-Shiow Chen, Wan-Chi Tsai

**Affiliations:** ^1^Department of Otolaryngology, Cathay General Hospital, Taipei City 106, Taiwan; ^2^Department of Otolaryngology, Sijhih Cathay General Hospital, New Taipei City 221, Taiwan; ^3^School of Medicine, Fu-Jen Catholic University, New Taipei City 242, Taiwan; ^4^Department of Hemato-Oncology, Chi-Mei Medical Center, Tainan City 710, Taiwan; ^5^Department of Medical Laboratory Science and Biotechnology, Kaohsiung Medical University, Kaohsiung 807, Taiwan; ^6^Department of Medical Imaging and Radiological Sciences, Kaohsiung Medical University, Kaohsiung 807, Taiwan; ^7^Center for Infectious Disease and Cancer Research, Kaohsiung Medical University, Kaohsiung 807, Taiwan; ^8^Graduate Institute of Medicine, College of Medicine, Kaohsiung Medical University, Kaohsiung 807, Taiwan; ^9^Institute of Medical Science and Technology, National Sun Yat-Sen University, Kaohsiung 804, Taiwan; ^10^Department of Medical Research, Kaohsiung Medical University Hospital, Kaohsiung 807, Taiwan; ^11^Institute of Cosmetic Science, Chia-Nan University of Pharmacy and Science, Tainan City 717, Taiwan

In the article titled “Anticancer Effects of* Salvia miltiorrhiza* Alcohol Extract on Oral Squamous Carcinoma Cells” [1], the name of the second author was given incorrectly as Kuo-Yu Hsuan. The author's name should have been written as Yu-Hsuan Kuo. The revised authors' list is shown above.
