# Supplementary material for: Anticancer Effects of Salvia miltiorrhiza Alcohol Extract on Oral Squamous Carcinoma Cells
Source: Evid Based Complement Alternat Med. 2017 Jan 29;2017:5364010. doi: 10.1155/2017/5364010 (PMC5303586; doi:10.1155/2017/5364010)
Supplement: Supplementary file 1 — Gingival tissue from healthy outpatients with the patients' consent was used as a source of NOK, which were maintained in Keratinocyte serum-free medium (Invitrogen, Carlsbad, CA) and used within ﬁve passages. [file 5364010.f1.pdf]

Anti-cancer Effects of *Salvia miltiorrhiza* Alcohol Extract on Oral Squamous Carcinoma Cells

Supplementary Information

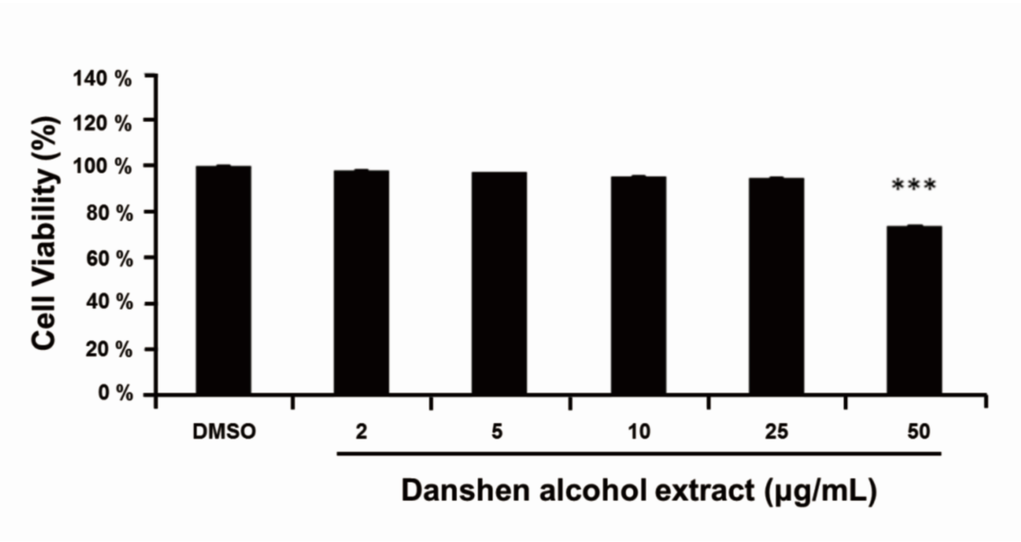

Supplementary Figure S1. NOK cell viability following treatment with Danshen alcohol extract. Cells were treated with concentrations of 0, 2, 5, 10, 25 or 50 μg/ml of Danshen alcohol extract for 24 hrs. MTT assay results are expressed as mean ± SEM (n=4). ***$p < 0.001$.

Table S1. Quantitative HPLC data for two major components in extracts from China- and Taiwan-grown Danshen plants.

Sample	Salvianolic acid B (mg/g)	Tanshinone IIA (mg/g)
CD1	86.05	3.42
CD2	101.07	3.04
CD3	85.60	0.08
TD1	121.82	4.94
TD2	100.46	3.44
TD3	95.10	0.02

CD: China Danshen; TD: Taiwan Danshen.
CD1 and TD1: 95% ethanol extract; CD2 and TD2: 1:1 water/ethanol extract; CD3 and TD3: water extract.

Table S2. DPPH radical scavenging data for extracts from China- and Taiwan-grown Danshen plants.

Sample	Scavenging Rate (%)				SC_{50}
	0.08 (mg/mL)	0.1 (mg/mL)	0.2 (mg/mL)	0.4 (mg/mL)	
CD1		30.57 ± 4.56	49.62 ± 3.03	66.61 ± 2.40	0.331
CD2	26.68 ± 0.97	34.17 ± 4.21	55.17 ±8.52		0.198
CD3	29.60 ± 4.04	38.47 ± 5.94	64.58 ± 8.60		0.132
TD1	42.00 ± 0.70	54.55 ± 1.38	71.20 ± 4.27		0.094
TD2		24.66 ± 4.44	38.90 ± 2.92	59.90 ± 0.99	0.311
TD3		32.28 ± 3.74	45.33 ± 5.16	63.99 ± 3.11	0.260
	10	20	40		SC_{50}
Trolox	25.39 ±0.35	44.13 ±1.96	91.35 ±0.80		0.022

Note: Trolox served as a positive control.
CD: China Danshen; TD: Taiwan Danshen.
CD1 and TD1: 95% ethanol extract; CD2 and TD2: 1:1 water/ethanol extract; CD3 and TD3: water extract.

Table S3: ABTS radical cation-scavenging activity in different extracts from China or Taiwan Danshen.

Sample	Scavenging Rate (%)				SC_{50}
	0.08 (mg/mL)	0.1 (mg/mL)	0.2 (mg/mL)	0.4 (mg/mL)	
CD1		26.35 ± 2.12	45.40 ± 2.83	62.61 ± 0.35	0.284
CD2		25.85 ± 0.75	46.66 ± 0.61	73.77 ± 0.71	0.248
CD3		26.86 ± 0.30	47.58 ± 1.93	81.31 ± 0.61	0.228
TD1		34.58 ± 1.89	55.70 ± 0.76	97.93 ± 0.11	0.197
TD2		21.51 ± 1.85	49.85 ± 1.85	87.78 ± 3.09	0.232
TD3		37.16 ± 2.91	47.82 ± 2.01	88.02 ± 2.49	0.223
	20	40	50	80	SC_{50}
Trolox	20.80± 0.87	47.60± 0.90	53.97± 1.02	94.25± 0.83	0.048

Note: Trolox served as a positive control.
CD: China Danshen; TD: Taiwan Danshen.
CD1 and TD1: 95% ethanol extract; CD2 and TD2: 1:1 water/ethanol extract; CD3 and TD3: water extract.
